# Supplementary material for: The interactome of intact mitochondria by cross-linking mass spectrometry provides evidence for coexisting respiratory supercomplexes
Source: Mol Cell Proteomics. 2017 Dec 8;17(2):216–32. doi: 10.1074/mcp.RA117.000470 (PMC5795388; doi:10.1074/mcp.RA117.000470)
Supplement: Supplemental Data [file supp_RA117.000470_133922_0_supp_23978_fzffwf.zip › spectra_annotation/mito_spectra_annotation/280-1-4-1-3-1.pdf]

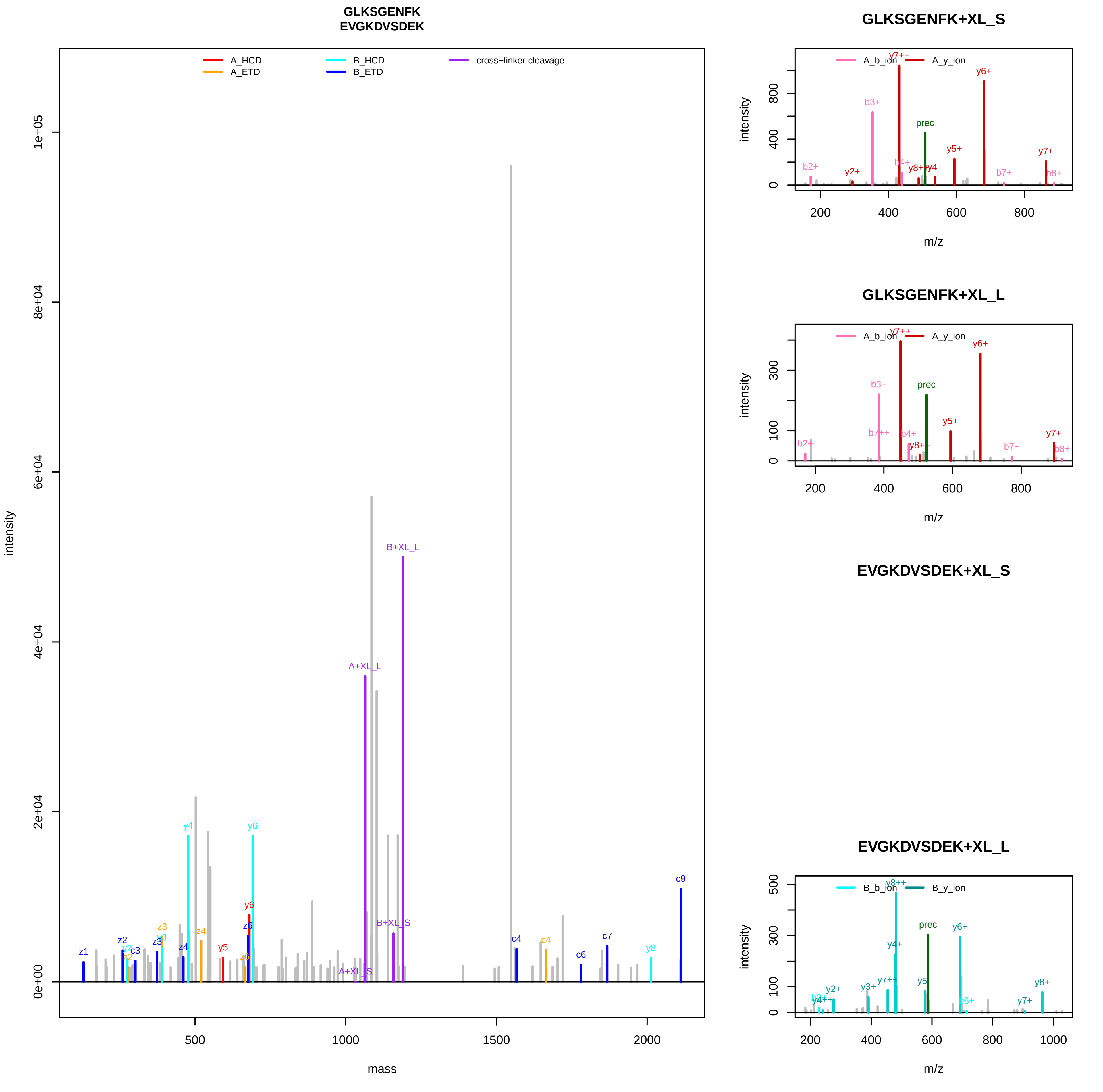
GLKSGENFK
EVGKDVSDEK
A_HCD
A_ETD
B_HCD
B_ETD
cross−linker cleavage
intensity
mass
0e+00
2e+04
4e+04
6e+04
8e+04
1e+05
500
1000
1500
2000
A+XL_L
B+XL_L
B+XL_S
A+XL_S
y4
y6
y5
z1
z2
z3
z4
z6
c3
c4
c6
c7
c9
y8
GLKSGENFK+XL_S
GLKSGENFK+XL_L
EVGKDVSDEK+XL_S
EVGKDVSDEK+XL_L
A_b_ion
A_y_ion
B_b_ion
B_y_ion
prec
m/z
b2+
b3+
b7+
y2+
y5+
y6+
y7+
y7++
y8++
y3+
y4+
y4++
y8+
b7++
